# Analysis of *APC* promoter 1B deletions in Russian families with familial adenomatous polyposis

**DOI:** 10.3389/fonc.2026.1834702

**Published:** 2026-08-19

**Authors:** Aleksey S. Tsukanov, Sergey I. Achkasov, Anna N. Loginova, Dmitry Yu. Pikunov, Vitaly P. Shubin, Aleksandra S. Monakhova, Anastasiia V. Kashchenko, Yulia M. Suvorova, Evgeny I. Klimuk, Konstantin V. Severinov

**Affiliations:** 1 Ryzhikh National Medical Research Centre for Coloproctology, Moscow, Russia; 2 Biotechnology campus LLC, Moscow, Russia

**Keywords:** *APC* promoter 1B deletion, familial adenomatous polyposis, founder effect, MLPA, whole genome sequencing

## Abstract

**Objective:**

Familial adenomatous polyposis (FAP) is a severe autosomal dominant hereditary cancer syndrome. Patients develop hundreds of adenomatous polyps throughout the colon with the risk of colorectal cancer, if untreated, approaching 100%. FAP is caused by pathogenic germline variants in the *APC* gene. Deletions in the *APC* 1B promoter cause FAP in a small subgroup of patients. Previous studies suggested that the *APC* promoter deletions in unrelated FAP patients from the US and Italy are identical and may thus have spread from a single founder. The aim of this study was to investigate whether a similar founder effect can be detected in the Russian population.

**Patients and methods:**

We performed whole-genome sequencing on five unrelated patients (three males and two females) with extensive (over 100) colon polyps, family history of FAP, and germline *APC* 1B promoter deletions previously detected by the multiplex ligation-dependent probe amplification (MLPA) and detected precise deletion boundaries.

**Results:**

The patients carried deletions in the *APC* 1B promoter ranging from ~3 to ~122 kbp. We found no association between the deletion size and either the age of the onset or severity of the disease. All deletions were unique and no identical deletion boundaries were observed. However, in four patients, the right deletion breakpoints fell into a 1 kbp region downstream of the 1B promoter. The right breakpoints of several deletions detected in FAP patients from other countries also fell into this narrow region.

**Conclusion:**

The *APC* 1B promoter deletions analyzed in this study had arisen independently and there is thus no evidence of a founder effect. Therefore, at least for the cohort of FAP patients with *APC* 1B promoter deletions studied here, WGS did not provide an added diagnostic benefit to MLPA aside from precisely determining the deletion breakpoints.

## Introduction

Familial adenomatous polyposis (FAP) is a hereditary cancer syndrome characterized by the development of hundreds, sometimes thousands, of adenomatous polyps in the colon. If proctocolectomy is not performed, the lifetime risk of colorectal cancer (CRC) approaches 100% (1). The incidence of FAP is approximately 1 in 10, 000 live births (2). The onset of the disease can occur in late teens, however, the diagnosis is made much later (3). In addition to colorectal cancer, patients with FAP may develop hepatoblastomas, medulloblastomas, as well as malignant tumors of the duodenum, stomach, and less often, in other organs (4). Depending on the population studied, germline pathogenic variants in the *APC* gene (Adenomatous Polyposis Coli, OMIM # 611731) occur in 70 to 90 percent of FAP patients (5, 6). While many FAP cases are familiar, about 20-30% are caused by *de novo APC* pathogenic variants (7).

*APC* is a tumor suppressor gene located on chromosome 5. It spans over 138, 000 bp and encodes a 2, 843-amino acid protein that negatively regulates the WNT signaling pathway. At the time of this writing, 2, 918 unique pathogenic and likely pathogenic (P/LP) variants in the *APC* gene have been described in the ClinVar database. Most of these variants are frameshifts (1594); 703 are nonsense, and 21 are missense mutations. Two frameshift *APC* variants, c.3927_3931del and c.3183_3187del, are the most common and are found in ~28% of patients (7).

A subset of known P/LP *APC* variants comprise intronic mutations and structural variations. Structural rearrangements in the *APC* gene have been observed in ~2% of FAP patients in the USA and ~14% in China (7, 8). These include large deletions of the entire gene and surrounding sequences, deletions and/or inversions of exons, and, infrequently, deletions of one of the two *APC* promoters, the 1B promoter. Snow et al. described an *APC* promoter 1B deletion in probands from seven US families with FAP. Using MLPA and haplotype analysis, they suggested that these deletions likely had arisen due to a “founder effect” (9). In a subsequent study, the authors precisely determined deletion coordinates using WGS in one patient and then used a PCR based approach to confirm the founder effect (10). Later, another founder 1B promoter deletion was identified in 3 families from Italy using a combination of MLPA (Multiplex Ligation-dependent Probe Amplification), PCR and Sanger sequencing (11).

Previously we identified *APC* promoter 1B deletions in three unrelated patients from Russia (6). However, whether these structural variants shared the same boundaries and thus could have also arisen due to a founder effect was not established. This study aims to fill this gap by analyzing whole-genome sequences (WGS) of three original patients and two additional unrelated individuals with *APC* 1B promoter deletions.

## Data and methods

From January 2018 to November 2022, 207 FAP patients were seen at the Ryzhikh National Medical Research Centre for Coloproctology. Five unrelated patients (3 males, 2 females), aged 28–36 at the time of diagnosis, were identified via the Local Registry of Hereditary Colorectal Cancer. Every patient had a germline large deletion of the *APC* 1B promoter revealed by MLPA. An 18-year-old son of one of the patients, who was also diagnosed with FAP, was also included in the study. Informed written consent was obtained from each participant. The study was conducted in accordance with the Declaration of Helsinki and was approved by the RNMRCC Ethics Committee.

### Whole genome sequencing and analysis

Genomic DNA was extracted using the magnetic bead-based sorption method (MGIEasy Magnetic Beads Blood Genomic DNA Extraction Kit, MGI) and subsequently used for the preparation of genomic libraries for sequencing. Library preparation was performed using a PCR-free protocol with enzymatic DNA fragmentation (MGIEasy FS PCR-Free Library Prep Set, 96 reactions (MIX), MGI). Prepared libraries were sequenced on the DNBSEQ-T7 sequencer (PE150) with a target median coverage of 30×.

Reads were passed to cutadapt 4.2 (12) for adapter removal (MGIEasy DNA Adapters) and trimming of low-quality read ends. Mapping to the GDC reference genome GRCh38.d1.vd1 was performed with bwa 0.7.17 (13). Duplicate marking was performed with Picard 2.27.5. Coordinates of insertion were identified using the Manta structural variant caller (14) and verified by manual expectation using the IGV browser.

Python scripts were utilized for searching for deletion-related sequences around breakpoints and similarity analyses. RepeatMasker was used for searching transposable elements (long interspersed nuclear elements (LINE), short interspersed nuclear elements (SINE) including Alu and MIR elements and long terminal repeat (LTR) elements) and low complexity sequences. A sequence logo was created using WebLogo (15).

## Results

**Figure 1:**
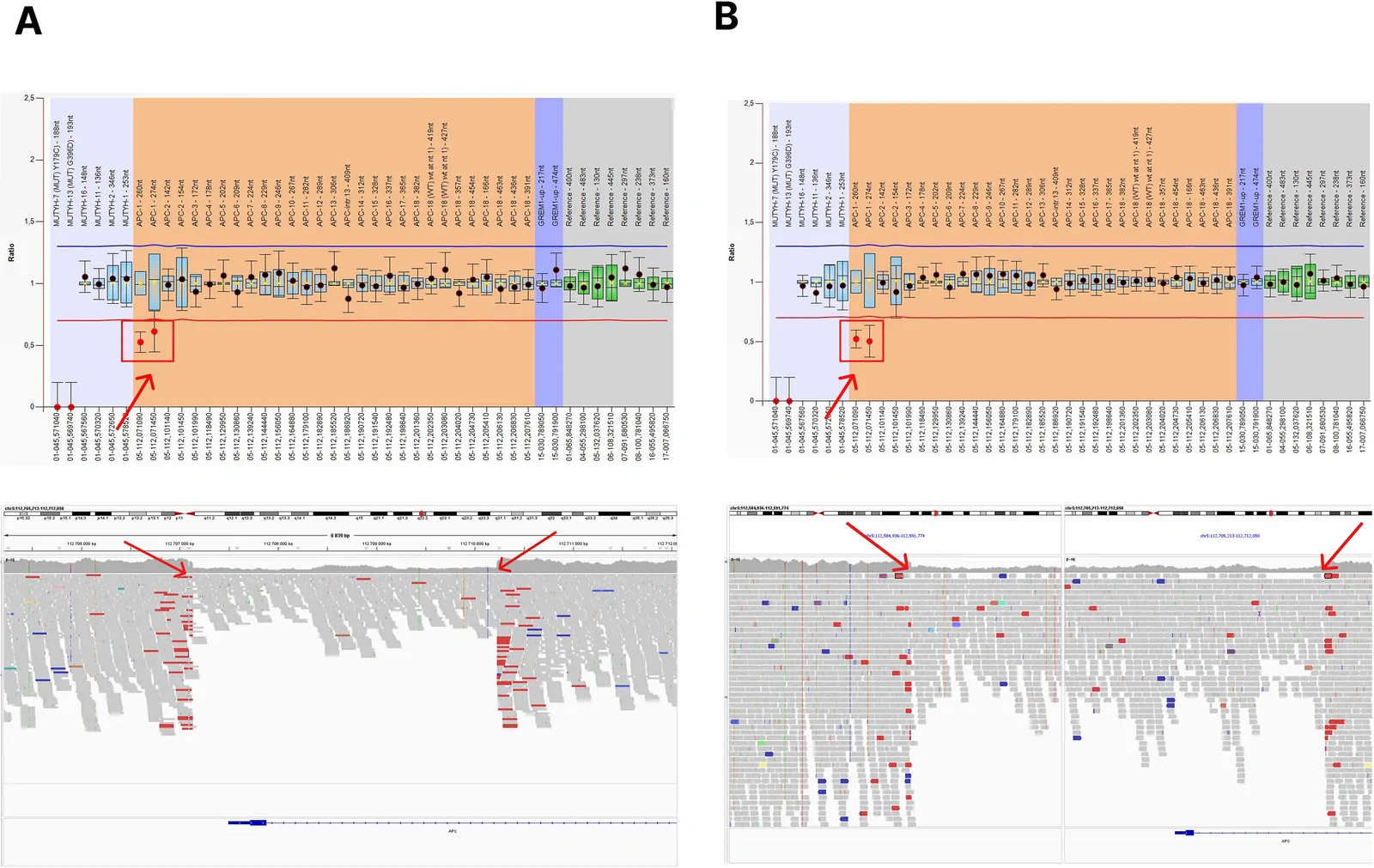
Comparison of the MLPA and WGS results for FAP patients with the smallest and the largest *APC* 1B promoter deletions. Above, the results of MLPA testing for two FAP patients from our cohort with the smallest (patient A966 - deletion size 3, 089 bp, **(А)** and the largest (patient A473 - deletion size 121, 907 bp, **(B)** *APC* 1B promoter deletions are shown. The red rectangles highlight MLPA signal indicating the presence of *APC* promoter 1B deletions. Below, IGV viewer screens of read alignment in regions affected by deletions in both patients are shown, red arrows indicate deletion breakpoints. The two IGV screens are not in scale to accommodate the different sizes of deletions.

Deletions of the *APC* 1B promoter have been identified by MLPA in three previously described (6) and two additional unrelated FAP patients recruited for this study. Since Sanger sequencing of the *APC* gene failed to reveal any P/LP variants, the *APC* 1B promoter deletions must have been the cause of the disease in all five patients. Whole genome sequencing of DNA prepared from whole blood samples was performed to define deletion boundaries. The genome of the son of one of the female patients, also diagnosed with FAP, was also sequenced. The results, summarized in **Table 1**, revealed that each unrelated patient harbored a distinct deletion ranging from 3, 089 bp to 121, 907 bp (**Table 1**). In our cohort the deletion size did not correlate with the age of the onset or severity of the disease. For example, FAP was diagnosed at age 36 in a patient with the smallest (3, 089 bp) deletion; age 28 in a patient with a 7283 bp deletion, and age 32 and in a patient with the largest (121, 907 bp) deletion. Further, patient A966 with the smallest deletion was diagnosed with FAP at the age of 36, while her son, who, expectedly, had the same deletion, was diagnosed when he was 18 years old. As can be seen from **Figure 1**, MLPA lacks the resolution to determine the sizes of *APC* 1B deletions, providing the same result for patients with the smallest (panel A) and the largest (panel B) deletions.

**Table 1:** Data on FAP patients with *APC* 1B promoter deletions.

Patient	Age of FAP diagnosis	Gender	Number of polyps/colorectal cancer (CRC) events	Number of affected relatives	Deletion coordinates (GRCh38, chr5)		Deletion size, bp
					Start	End	
A473	32	F	100+/3 CRC	4	112, 588, 683	112, 710, 589	121, 907
A836	35	M	100+/1 CRC	3	112, 692, 168	112, 710, 894	18, 727
A853	28	M	100+	1	112, 702, 822	112, 710, 104	7, 283
А875	34	M	100+	2	112, 690, 128	112, 717, 043	26, 916
A966	36	F	100+/3 CRC	6	112707136	112710224	3, 089
А1046 (the son of A966)	18	M	100+	6	112707136	112710224	3, 089

**Figure 2:**
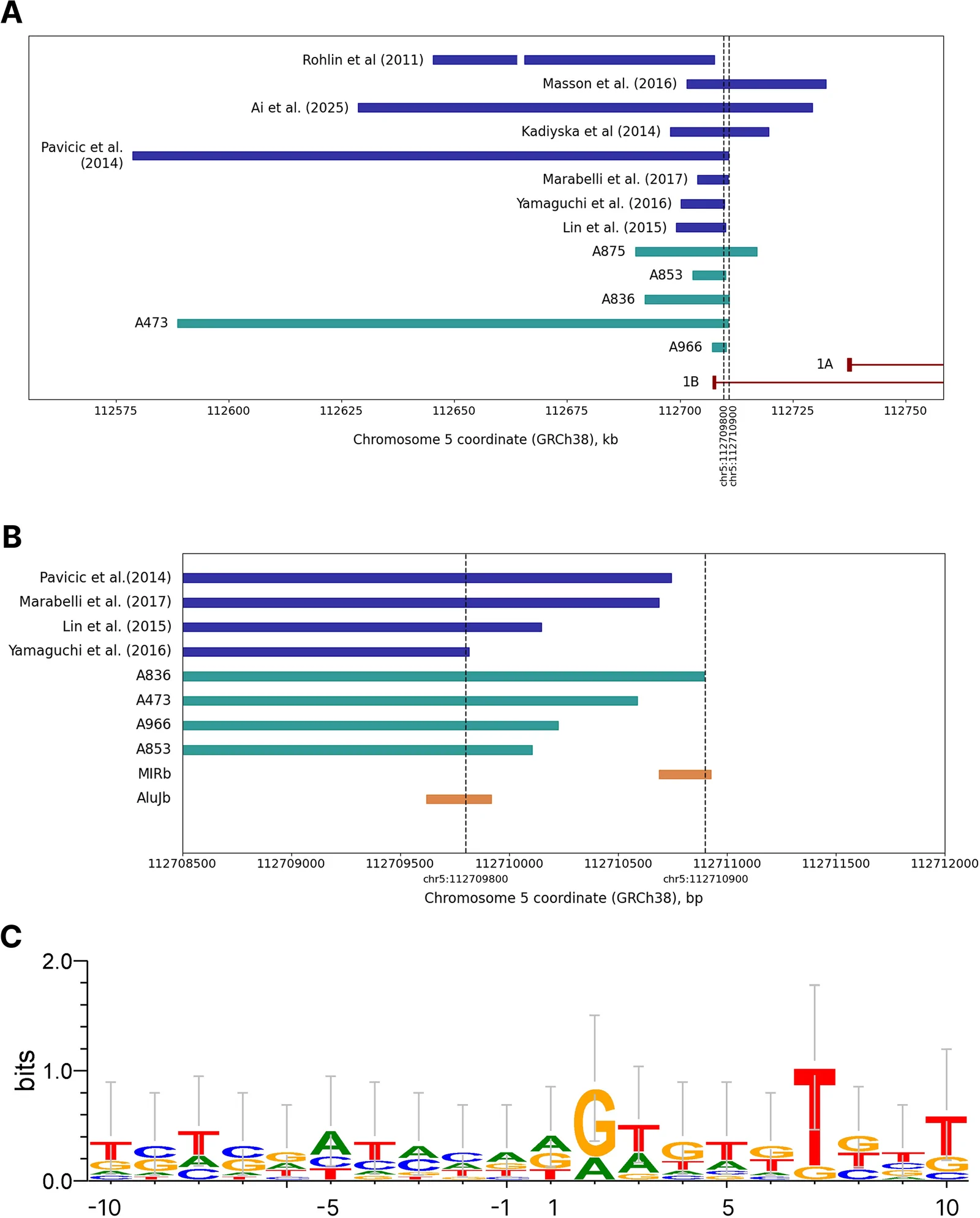
Analysis of a 1 kbp interval of *APC* 5’ UTR that contains right breakpoints of multiple deletions causing FAP. **(A)** Schematic representation of *APC* 1B promoter deletions with known boundaries. Chromosome 5 coordinates are shown below. The positions of rightward-oriented 1B and 1A *APC* promoters are indicated. The red lines indicate *APC* transcripts. Known *APC* 1B promoter deletions are shown above as thick horizontal lines: deletions from this study are shown in cyan and marked by patient’s ID; deletions from other studies - in dark blue. The 1 kbp interval that harbors right breakpoints of multiple deletions is located in between black dashed lines. **(B)** The 1 kbp interval is expanded with the positions of repetitive elements that it contains/overlaps with are shown. **(C)** WebLogo representation of sequence conservation within 10 nucleotide intervals upstream and downstream of right breakpoints of eight deletions falling within the 1 kbp interval.

Although every deletion in our study was unique, the right breakpoints of four deletions fell within an ~1 kbp chr5:112, 709, 800-112, 710, 900 interval. The left breakpoints varied widely, as expected given the different deletion sizes. The interval where the right breakpoints are enriched is located downstream of the 1B promoter transcript start point in the beginning of a long non-coding untranslated region of the *APC* gene (**Figure 2A**). The 1A promoter, located much closer to the *APC* gene, was not affected by deletions from our set.

We compared deletions observed in Russian patients with *APC* 1B promoter deletions with precisely established boundaries observed in patients from different countries (**Table 2**). In a combined set of 13 unique deletions, 8 had their right breakpoints within the 1 kbp interval downstream of the 1B promoter (**Figure 2A**). Deletions that were attributed to founder effects in the US and Italian families, as well as a deletion observed in a family from Finland and another one found in a single FAP patient from Japan, also had their right breakpoints inside this interval.

**Table 2:** *APC* promoter 1B deletions detected in previous studies.

Country	GRCh38*, chr5		Size (kb)	Cohort	Publication	Method
	Start	End				
Japan	112, 700, 228	112, 709, 815	9.5	Single patient	(16)	WGS, PCR & Sanger
USA (founder)	112, 699, 127	112, 710, 148	11	7 families	(9, 10, 17)	Target NGS + PCR & Sanger
Italy (founder)	112, 703, 831	112, 710, 688	7	3 families	(11)	MLPA + array-CGH + PCR & Sanger
Sweden	112, 665, 480	112, 707, 631	total 61	One family (two adjacent deletions)	(18)	CNV detection + PCR & primer walking
	112, 645, 201	112, 663, 968				
Finland	112, 578, 666	112, 710, 744	132	One family	(19)	Long range PCR + primer walking
Bulgaria	112, 697, 798	112, 719, 689	21.8	One family	(20)	MLPA + array-CGH + long range PCR + Sanger
China	112, 628, 696	112, 729, 368	100	One family	(21)	NGS + Sanger
Australia	112, 701, 437	112, 732, 406	31	One family	(22)	Cytogenomic microarray + qPCR

*Coordinates from original studies were transferred to the reference genome GRCh38 using LiftOver.

It appears that a compact interval downstream of the *APC* 1B promoter may represent a hotspot that induces formation of deletions leading to FAP. To reveal the possible mechanism(s) leading to apparently frequent formation of deletions within the 1 kbp interval, we checked several hypotheses. To examine whether deletions were induced by the presence of repetitive elements, we analyzed the 1 kbp region using RepeatMasker. A copy of a divergent MIRb/SINE element occupies the last 200 bp of the 1 kbp interval (**Figure 2B**, this element was previously observed and discussed in (11)). There is also an AluJb/SINE element overlapping with the beginning of the 1 kbp interval (**Figure 2B**). Since multiple deletion breakpoints are located in the central part of the 1 kbp element, these repetitive sequences are unlikely to promote deletion formation. To examine whether deletion breakpoints falling into the 1 kbp region share a common motif, similarity analysis of sequences around the right breakpoints in the four Russian patients and in four deletions from other studies was performed. No evidence of conservation was revealed (**Figure 2C**), suggesting that local context played no role in deletion formation. To determine if the *APC* promoter deletions were caused by microhomology-mediated end-joining, the breakpoint sequences for each deletion were examined separately. The only deletion that contained apparent microhomology (TGTGT, patient А875) had its right breakpoint outside of the apparent hotspot interval. Analysis of this deletion with RepeatMasker revealed that its right breakpoint fell within an AluSx3 (SINE/Alu) element in the 5’ UTF of the *APC* gene. Overall, we conclude that while the 1 kbp region downstream of the 1B *APC* promoter is enriched in deletion breakpoints, the reasons for this enrichment remain to be determined.

## Discussion

Multigene hereditary−cancer panels are widely used in diagnostic settings. However, they cannot detect structural variations, leaving some cases unexplained. Lee et al. reported that MLPA identified large *APC* deletions or duplications in 7 out of 29 (24.1%) FAP patients who obtained negative results during panel testing (23). Though lower frequencies of structural variations in FAP patients were reported in other studies (10.3% in Hungary (24), 8.7% in Brazil (25), and 3.2% in Germany (26)), routine usage of MLPA for patients with negative panel testing results is clearly beneficial.

However, MLPA does not precisely determine the breakpoints and thus does not allow to establish exact deletion/duplication sizes, which is required for identification of founder events. Complementing MLPA with whole-genome sequencing and/or long−read sequencing technologies in diagnostic workflows would enhance detection and enable accurate characterization of structural variants.

Using MLPA, we previously found *APC* deletions/duplications in 9.6% (8/83) of FAP patients (6). Three cases (each with a family cancer history) involved *APC* 1B promoter deletions. In similar cases in the United States and Italy such deletions were attributed to founder effects (9, 11). To explore whether *APC* 1B promoter deletions in Russian patients were also due to a founder variant, we here performed WGS analysis of five unrelated FAP patients with 1B deletions. We reasoned that if the founder effect was confirmed, a straightforward real−time PCR test could have been designed to speed up screening and reduce its cost. However, all deletions were unique, and so there is no evidence of a founder effect in our cohort. Since deletion sizes did not affect the severity or prognosis of the disease in our patients (including in two members of one family with the same deletions), MLPA appears to be sufficient for routine DNA diagnostics of FAP in patients with 1B promoter deletions. A much larger cohort study would be necessary to determine whether any of deletions identified in this study are founder mutations characteristic for the Russian population and to investigate in greater detail the relationship between deletion sizes and phenotypes. However, this may be complicated by the rarity of the *APC* 1B promoter deletions.

## Data Availability

The raw sequencing data for this study are not publicly available due to privacy issues and legislation restriction. Requests to access the datasets should be directed to the corresponding author individually.
